# Performance of Large Language Models in Metabolic Bariatric Surgery: a Comparative Study

**DOI:** 10.1007/s11695-025-08418-y

**Published:** 2025-12-11

**Authors:** Hassan El-Masry, Mohamed Yasser El-mezayen, Bothaina Farouk, Abdelrahman M. Tawfik, Passant Saeed Sharsher, Basma Hesham Mohamed, Ahmed Abo Elmagd, Ali Khammas, Abdelrahman Nimeri, Ricardo V Cohen, Ahmed Abokhozima

**Affiliations:** 1 Alexandria University, Alexandria, Egypt; 2 MIAX Research Lab, Alexandria, Egypt; 3 Rashid Hospital, Dubai, United Arab Emirates; 4 Brigham and Women’s Hospital, Harvard Medical School, Boston, USA; 5 The Center for the Treatment of Obesity and Diabetes, Hospital Alemão Oswaldo Cruz, São Paulo, Brazil

**Keywords:** Large language models, Artificial intelligence, Medical education, ChatGPT, Gemini, Surgical training

## Abstract

**Background:**

The rapid integration of Large Language Models (LLMs) into healthcare necessitates a rigorous evaluation of their performance in specialized medical fields. In metabolic bariatric surgery (MBS), LLMs have the potential to revolutionize education and clinical support, yet their accuracy and reliability are not well-established. This study provides a critical assessment of the capabilities of current LLMs in the context of MBS.

**Methods:**

This cross-sectional validation study assessed the performance of six LLMs (ChatGPT-3.5, ChatGPT-4o, Gemini, Copilot, GROK, and DeepSeek) in answering 100 evidence-based binary and multiple-choice questions related to MBS. Questions were constructed from international guidelines and categorized into six thematic domains. Expert consensus answers served as the reference standard, with inter-rater reliability measured using Fleiss’ κ. Model outputs were scored for accuracy. Comparisons across LLMs were first assessed using an overall test for differences between multiple related groups. Pairwise comparisons were then conducted between LLMs to identify specific differences in performance.

**Results:**

Across the dataset, the mean number of correct LLM responses per question was 3.9 (SD = 1.8). ChatGPT-4o achieved the highest accuracy (66.0%), while DeepSeek recorded the lowest (60.0%). Accuracy varied across domains, highest for indications/contraindications (78.7%) and complications/management (68.0%), and lowest for preoperative preparation (52.0%) and postoperative care (58.4%). Binary questions yielded higher accuracy (69.1%) than multiple-choice questions (62.0%). Inter-expert reliability was substantial (κ = 0.742, 95% CI: 0.71–0.77). Agreement between LLMs and experts ranged from fair (DeepSeek κ = 0.349) to moderate (ChatGPT-4o κ = 0.446). No significant accuracy differences were detected across models (Friedman test, *p* = 0.662).

**Conclusion:**

LLMs represent a promising, yet imperfect, adjunct in MBS education. Their utility is currently limited by inconsistencies in accuracy, particularly in areas requiring nuanced clinical judgment. While these models can supplement traditional learning resources, they are not yet a substitute for expert clinical guidance. This study underscores the need for continued refinement and validation of LLMs to ensure their safe and effective integration into clinical practice.

**Supplementary Information:**

The online version contains supplementary material available at 10.1007/s11695-025-08418-y.

## Key Points

LLMs show moderate accuracy in bariatric surgery education, strongest in guideline-based domains.

Newer models (ChatGPT-4o, Gemini, Copilot) performed slightly better, but gains were modest.

Accuracy was higher for binary than multiple-choice questions.

**Supplementary Information:**

The online version contains supplementary material available at 10.1007/s11695-025-08418-y.

## Background 

 Large Language Models (LLMs) are a form of artificial intelligence designed to understand and produce human-like text [1]. Since the public release of ChatGPT by OpenAI in November 2022 (https://chat.openai.com) [2], these models have gained widespread attention. They can perform complex language tasks such as answering queries, summarizing information, rephrasing content, and translating languages, often with a fluency and accuracy that closely mirrors human performance [3]. Beyond ChatGPT, several other powerful LLMs have emerged. Claude, developed by Anthropic, emphasizes safe and interpretable AI responses [4]. DeepSeek, a Chinese-developed LLM, focuses on code generation and language tasks with competitive multilingual performance [5]. Meanwhile, Google’s Gemini (formerly Bard) integrates language understanding with real-time search capabilities, offering responses grounded in current web data [6].

While the answers generated by LLMs may sometimes be misleading, their increasing use by clinicians and patients highlights the importance of systematically evaluating their performance. The application of LLMs in medical education is a rapidly growing field of research, with studies exploring their use in various specialties, from internal medicine to radiology [7, 8]. In the context of metabolic bariatric surgery (MBS), LLMs offer promising applications for both surgeons and patients. They may assist in preoperative education, streamline postoperative care instructions, and support clinical decision-making by synthesizing evidence-based guidelines [9]. However, the unique complexities of surgical decision-making, which often involve a combination of evidence-based knowledge and nuanced clinical judgment, present a significant challenge for these models. Previous studies have highlighted the potential of LLMs in surgery, but have also raised concerns about their accuracy and reliability [10, 11].

In our study, we adopt a practical approach that involves assessing how accurately LLMs respond to a standardized set of evidence-based binary and multiple-choice questions (MCQs), comparing their selected answers against experts’ responses. This method provides a quantifiable way to gauge their medical reasoning and factual consistency, offering insight into their potential utility in clinical education, decision support, and patient engagement. This study is framed within the broader context of the Technology Acceptance Model (TAM), which posits that the perceived usefulness and ease of use of a technology are key determinants of its adoption [12]. By evaluating the accuracy of LLMs, we are, in effect, assessing a critical aspect of their perceived usefulness in a clinical setting.

## Methods

This study was designed as a cross-sectional validation study aimed at assessing the performance of six LLMs in providing accurate responses to a structured set of bariatric surgery questions. The evaluation was performed at the level of individual question–answer pairs, with each LLM tested on the same fixed set of items under standardized conditions.

### Questions Acquisition

The questions used in this study were developed by one of the co-authors, A.A., a metabolic bariatric surgeon with over 15 years of clinical and academic experience in the field (sup. 1). The questions were carefully constructed to reflect evidence-based guidelines and international recommendations, particularly those established by the International Federation for the Surgery of Obesity and Metabolic Disorders (IFSO) [13–17]. It is important to acknowledge that the development of the question set by a single author, while an expert in the field, represents a potential limitation. To mitigate this, the questions were reviewed by the expert panel for clarity and relevance prior to their use in the study. To ensure comprehensive coverage of the bariatric surgical pathway, the items were organized into six thematic categories: surgical techniques and procedures, indications and contraindications, effects and outcomes, preoperative preparation, postoperative care and recommendations, and complications with their management. This thematic structuring provided a balanced representation of the key domains of bariatric practice and maintained alignment with current best practices and clinical relevance [18, 19].

### Experts’ Answers Acquisition

To establish a reference standard for evaluating the LLMs-generated responses, the questions were first drafted and with correct responses by the question’s author (A.A.). To then establish a gold reference standard for evaluating the LLMs-generated responses, the questions were distributed via a Google Form to a panel of three MBS experts: R.C., A.K., and A.N. Each expert independently provided their answers based on current evidence and clinical best practices. The individual responses were then reviewed collectively to identify areas of concordance and discrepancy. Through a consensus process involving structured discussion and clarification, a final set of agreed-upon reference answers was established. The degree of agreement among the experts was documented to assess inter-rater reliability and to ensure the robustness of the reference standard used for comparison.

### LLMs’ Answers Acquisition

Each of the six LLMs under evaluation, ChatGPT-3.5, ChatGPT-4o, Grok 3, Gemini 1.5, Copilot (GPT-4), and DeepSeek V3 was prompted with the same fixed set of 100 bariatric surgery questions, which were provided by A.A. To ensure comparability, all models were accessed in their official public interfaces under standardized conditions between May and June 2025. For each question, a uniform input format was used, consisting solely of the question text without additional context or clarifying prompts, in order to minimize bias introduced by user–model interaction. The models were instructed to provide concise, evidence-based answers without references or extended explanations. All responses were exported, anonymized, and stored in a secure dataset. The answers were subsequently reviewed and scored against the reference standard.

### Statistical Analysis

Statistical analysis was conducted to evaluate the performance of the LLMs across the six thematic domains. Accuracy was first assessed by comparing each LLM’s responses against the reference standard established by the question author (A.A.), with results summarized using descriptive statistics (percentages and 95% confidence intervals). To validate the robustness of this reference standard, inter-rater reliability among the three bariatric surgery experts (R.C., A.K., and A.N.) was assessed using Fleiss’ kappa (κ). Their final consensus was considered the gold standard. Agreement between this expert consensus and the A.A.-defined reference standard was quantified to confirm consistency. Agreement between each LLM and the expert consensus gold standard was then measured using κ statistics for categorical data. A κ ≥ 0.75 was interpreted as excellent, 0.40–0.74 as moderate to good, and < 0.40 as poor agreement.

Comparisons of overall LLM performance were performed using the Friedman test. Where significant, post-hoc pairwise comparisons were conducted with Wilcoxon signed-rank tests and Bonferroni correction (adjusted α = 0.0033). McNemar’s test was used to assess paired differences in correctness patterns between models, while Jaccard similarity coefficients quantified the overlap of errors across models. Differences in performance between binary (yes/no, true/false) and multiple-choice (≥ 3 options) questions were analyzed using paired t-tests. All analyses were conducted in R version 4.4.2, with a two-tailed p-value < 0.05 considered statistically significant.

## Results

Across the 100 questions, the number of LLMs providing a correct response to each question ranged from 0 to 6 (mean = 3.9, SD = 1.8). Full agreement among all six models, where each provided the correct answer, was observed in 28 questions, most often on straightforward topics such as eligibility criteria (for example, Question 11: ‘Are amputees eligible for bariatric surgery?’). Among the six models, ChatGPT-4o achieved the highest accuracy (66.0%), while DeepSeek recorded the lowest accuracy (60.0%) (Table 1).

**Table 1:** Large language model-specific accuracies (percentage of correct answers)

Model	Accuracy (%)	95% Confidence Interval
ChatGPT-4o	66.0	56.7–74.4
Gemini	65.0	55.7–73.6
Copilot	65.0	55.7–73.6
GROK	64.0	54.7–72.7
ChatGPT-3.5	61.0	51.7–69.8
DeepSeek	60.0	50.7–68.8

The 100 questions were categorized into six thematic groups to facilitate analysis. Surgical techniques and procedures comprised the largest category (*n* = 30), including topics such as bougie size and limb lengths. Indications and contraindications accounted for 15 questions, addressing issues like BMI guidelines and autoimmune diseases. Another 15 questions focused on effects and outcomes, such as the impact of MBS on type II diabetes and Hyperlipidemia resolution. Preoperative preparation was covered by 5 questions, including diet protocols and medication cessation. Postoperative care and recommendations formed a substantial category with 25 questions, encompassing areas such as vitamin supplementation and activity resumption. Finally, 10 questions addressed complications and their management, including topics like leak management and dumping syndrome *(sup 2)*. For the accuracy of LLMs across these thematic groups, mean performance was as follows: surgical techniques and procedures, 62.3% (range: 58.3–66.7%); indications and contraindications, 78.7% (range: 73.3–86.7%); effects and outcomes, 60.0% (range: 53.3–66.7%); preoperative preparation, 52.0% (range: 40.0–60.0%); postoperative care and recommendations, 58.4% (range: 52.0–64.0%); and complications and management, 68.0% (range: 60.0–80.0%) (Table 2).

**Table 2:** Accuracy of large language models among six thematic groups of questions

Group of questions	Number of questions (*n*)	Mean accuracy (%)	Range (%)
Surgical techniques and procedures	30	62.3	58.3–66.7
Indications and contraindications	15	78.7	73.3–86.7
Effects and outcomes	15	60.0	53.3–66.7
Preoperative preparation	5	52.0	40.0–60.0
Postoperative care and recommendations	25	58.4	52.0–64.0
Complications and management	10	68.0	60.0–80.0

### Inter-Expert Reliability (A Gold Standard Comparison)

**Fig. 1:**
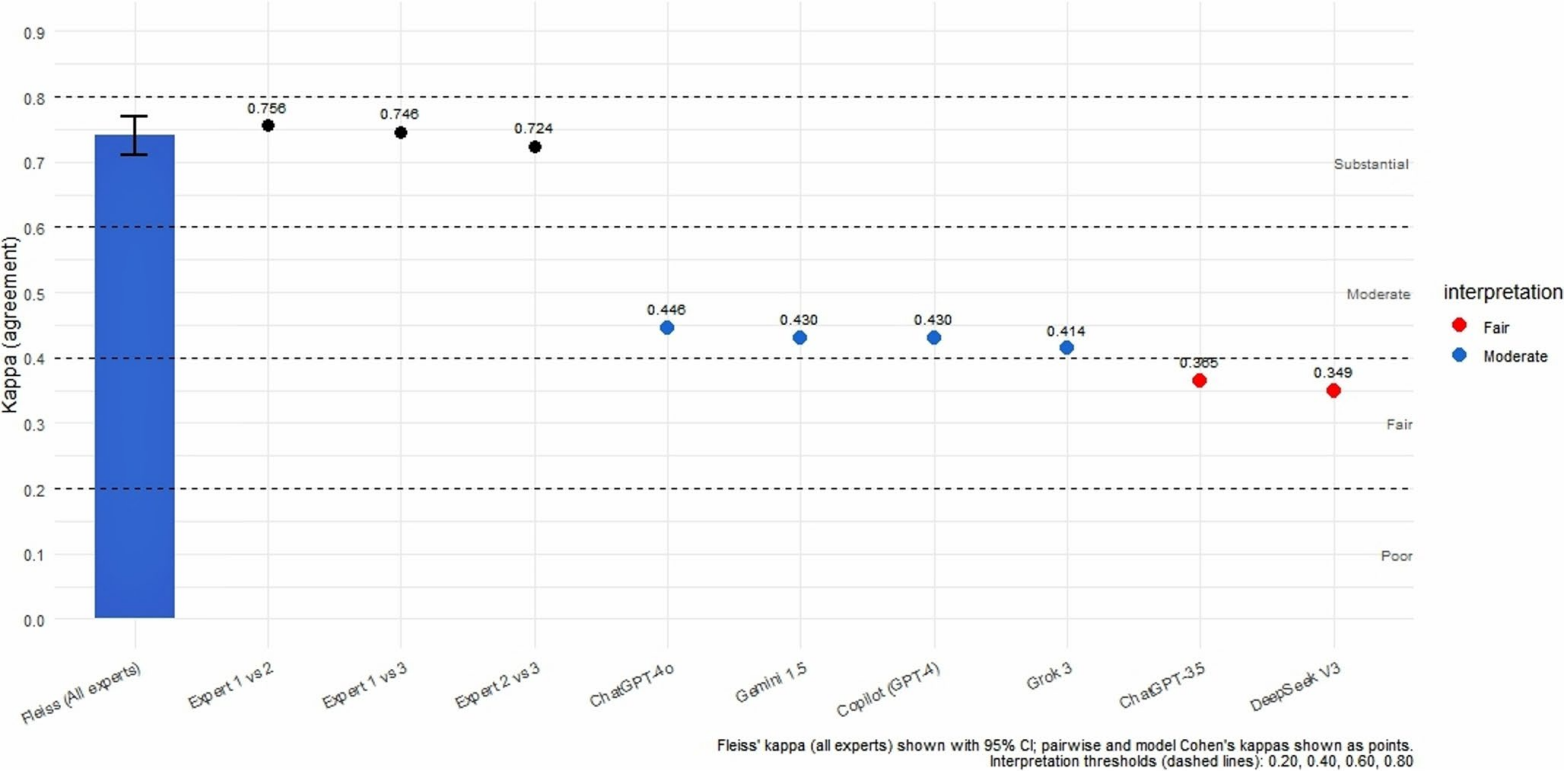
Agreement of Large Language Models (LLMs) with expert consensus in bariatric surgery questions

Inter-expert reliability was substantial, with Fleiss’ kappa of 0.742 (95% CI: 0.71–0.77). Pairwise comparisons showed high consistency among the experts: Expert 1–2 = 0.756, Expert 1–3 = 0.746, and Expert 2–3 = 0.724. When comparing each AI model to the expert gold standard, chance-adjusted agreement (Cohen’s kappa, with an expected agreement of 0.386 based on the average number of options per question) ranged from fair to moderate. ChatGPT-4o demonstrated the highest agreement (κ = 0.446, moderate), followed closely by Gemini and Copilot (κ = 0.430 each, moderate) and GROK (κ = 0.414, moderate). ChatGPT-3.5 (κ = 0.365) and DeepSeek (κ = 0.349) showed only fair agreement (Fig. 1).

The Friedman test indicated no significant overall differences in accuracy across the six models (χ²(5) = 3.25, *p* = 0.662). Post-hoc pairwise Wilcoxon signed-rank tests with Bonferroni correction (adjusted α = 0.05/15 = 0.0033) confirmed no significant differences (all adjusted *p* > 0.05). McNemar’s tests for paired model comparisons also showed no significant differences in correctness patterns (all *p* > 0.05; e.g., ChatGPT-4o vs. DeepSeek: *p* = 0.146; ChatGPT-3.5 vs. DeepSeek: *p* = 0.823).

### Difficulty and Error Pattern Analysis

Thirty-three questions were answered incorrectly by ≥ 3 models (models_correct_count ≤ 3), representing 33% of the dataset. These difficult questions clustered in postoperative care (*n* = 12, e.g., Question 32: caffeine avoidance timing, 0 correct), preoperative preparation (*n* = 5, e.g., Question 67: mandatory diet protocol, 0 correct), and surgical techniques (*n* = 8, e.g., Question 87: Roux limb length in RYGB, 2 correct). Errors often involved ambiguous clinical scenarios or guideline-specific details (e.g., Questions 10, 17, 27 all had 0 correct).

Error overlap between models was assessed via Jaccard similarity (proportion of shared incorrect questions relative to union). Similarities were moderate (mean = 0.52), highest between Gemini and Copilot (0.61) and lowest between ChatGPT-4o and DeepSeek (0.45), suggesting some shared weaknesses but model-specific error profiles.

### Comparative Performance of Large Language Models on Binary and Multiple-Choice Question Formats

Performance was compared between binary (*n* = 50, e.g., yes/no, true/false) and MCQs (*n* = 50, ≥ 3 options) (Table 3). Mean accuracy was higher for binary (69.1%) than MCQ (62.0%). LLMs generally performed better on fact-recall binary questions than reasoning-heavy MCQs, though differences were not statistically significant (paired t-test for overall, *p* = 0.128).

**Table 3:** Accuracy of large language models on binary vs. multiple-choice questions

Model	Binary Accuracy (%)	MCQs Accuracy (%)
ChatGPT-4o	72.3	67.4
Gemini	72.3	63.0
Copilot	68.1	67.4
GROK	70.2	63.0
ChatGPT-3.5	72.3	56.5
DeepSeek	63.8	60.9

## Discussion

The integration of technology into medical education is rapidly transforming how surgical trainees acquire knowledge and skills. MBS is a particularly dynamic field, characterized by a high volume of ongoing research and innovation. As junior surgeons often face challenges in accessing consistent, high-quality educational resources, there is a growing need for scalable, evidence-based learning tools. LLMs such as ChatGPT, DeepSeek, and Copilot are revolutionizing the way information is accessed and utilized in medical education.

These models, powered by advanced transformer architectures, are capable of understanding and generating human-like text, making them valuable tools for learning, revision, and clinical decision support. Our study aims to evaluate the accuracy and reliability of LLMs in responding to a standardized set of evidence-based binary and MCQs relevant to MBS. By benchmarking LLMs performance against established clinical guidelines and expert consensus, we seek to assess their potential as supplementary educational tools for surgical trainees with limited experience.

E-learning has proven particularly effective for young surgeons, as it provides structured, flexible, and interactive opportunities to build foundational knowledge and practice clinical decision-making before entering the operating room [20]. For trainees who face limited operative exposure due to time, resources, or patient safety constraints, web-based platforms offer a scalable and individualized means of reinforcing core surgical principles. The ability to revisit complex cases, receive immediate feedback, and adapt learning to personal pace ensures that junior surgeons can progress more efficiently toward competency. Importantly, as surgical technologies advance rapidly, young surgeons must also learn faster to keep pace, highlighting the need for e-learning systems that can deliver both breadth and depth of training material in real time [21].

The rapid evolution of artificial intelligence, and specifically LLMs, represents the next step in this educational transformation and e-learning methods. Recent evaluations underscore their potential as dynamic educational tools. For instance, our study assessed the performance of six LLMs in answering 100 MBS questions. The six models collectively achieved a mean of 3.9 correct responses per question, with full agreement among all six models in 28 instances, most often in straightforward domains. LLMs performed best on questions related to indications, contraindications, and complications, while showing weaker accuracy in preoperative preparation. Building on this, Samaan et al. were the first to evaluate ChatGPT in the bariatric surgery context, focusing on how reliably it could answer patients’ questions. They demonstrated that ChatGPT achieved an excellent accuracy rate, exceeding 90% for topics such as preoperative preparation and postoperative lifestyle changes [22].

Beyond overall accuracy rates, our study also explored the agreement between expert assessments and model outputs. Inter-expert reliability was substantial, with Fleiss’ kappa of 0.742 (95% CI: 0.71–0.77), confirming a high level of consistency among expert surgeons. Pairwise comparisons further supported this finding, with kappa values consistently above 0.72. When benchmarked against this expert-derived gold standard, LLMs demonstrated chance-adjusted agreement ranging from fair to moderate. ChatGPT-4o achieved the highest agreement (κ = 0.446), followed closely by Gemini, Copilot, and GROK. In contrast, ChatGPT-3.5 and DeepSeek demonstrated only fair levels of concordance.

ChatGPT-4o, Gemini, and Copilot clustered at the top with moderate agreement, while ChatGPT-3.5 and DeepSeek lagged at only fair levels. This indicates that newer-generation LLMs (particularly transformer-based with larger training corpora) do indeed outperform earlier ones, but the margin is not dramatic, which reinforces the idea that no single model is clearly superior in bariatric surgery education, even though some are incrementally stronger. Complementing these observations, Lee et al. evaluated ChatGPT-4, Bard, and Bing in generating clinician-level bariatric surgery recommendations and found substantial variability across models. ChatGPT-4 produced the highest proportion of appropriate responses (85.7%), compared with Bard (74.3%) and Bing (25.7%), although Bard offered better readability. Their findings highlight that while LLMs can deliver clinically relevant guidance, accuracy and usability remain model-dependent, underscoring the need for continued clinician oversight [23].

The way LLMs responded varied considerably across thematic domains. Accuracy was highest in indications and contraindications (78.7%) and complications and management (68.0%), reflecting the relative clarity and consistency of evidence-based guidelines in these areas. For example, questions about eligibility thresholds, comorbidity-based indications, or the management of well-defined complications such as leaks and dumping syndrome yielded higher consensus across models. These findings suggest that LLMs are more reliable when handling well-established, guideline-driven content with limited interpretive variability [24–26]. This aligns with previous research that has shown that LLMs are more adept at retrieving factual information than at engaging in complex clinical reasoning [27].

By contrast, performance was less accurate in preoperative preparation (52.0%) and postoperative care and recommendations (58.4%). Preoperative preparation often varies, whereas postoperative care is largely guided by well-known recommendations [13–17]. These cover areas such as diet sequencing, activity resumption, and medication adjustments. Despite being standardized in principle, differences in interpretation and application across settings likely contributed to inconsistent model responses and lower agreement with experts.

To further investigate performance patterns, accuracy was compared across binary and MCQs. Mean accuracy was higher for binary items (69.1%) than for MCQs (62.0%), which demonstrates LLMs generally performed better on fact-recall binary questions than on reasoning-heavy MCQs [28], though differences were not statistically significant (*p* = 0.128). This distinction mirrors the structure of surgical knowledge: binary responses resemble the immediate judgments made during intraoperative inspection (e.g., “Is perfusion adequate? Is a staple line intact?”), whereas MCQs parallel the layered reasoning required to interpret operative figures, imaging, and technical variations. In line with this, Mahjoub et al. recently demonstrated the accuracy of ChatGPT-4 in identifying six surgical procedures from standard illustrations. ChatGPT-4 correctly recognized only the adjustable gastric banding, misclassifying the others [29]. These findings parallel the drop in accuracy observed in MCQs, reinforcing that figure-based, reasoning-intensive assessments demand higher cognitive integration than factual recognition [28, 30].

In a separate effort to assess the reliability of ChatGPT-4 in bariatric surgery, Jazi et al. conducted a multinational survey involving thirty expert bariatric surgeons from twenty-four countries [31]. Ten diverse patient scenarios, ranging from straightforward to complex, were developed, and ChatGPT-4 was queried in two independent sessions to assess reproducibility. The model’s recommendations aligned with expert consensus in only 30% of cases, and it suggested surgery in 60% of patients compared with 90% deemed eligible by experts. Furthermore, 40% of scenarios yielded inconsistent answers between the two sessions, underscoring the variability and limited reliability of ChatGPT-4 in clinical decision-making.

LLMs show clear promise as adjuncts in bariatric surgery education, but they cannot yet substitute for expert guidance. Their incremental improvements across generations highlight the trajectory of progress, but variability in accuracy and reproducibility underscores the need for careful integration. From an educational standpoint, this means LLMs should be regarded as supplementary tools, valuable for reinforcing knowledge, stimulating clinical reasoning, and widening access to information, yet always embedded within structured curricula and supervised by experienced clinicians.

This study has several limitations that should be acknowledged. First, the question set was developed by a single author, which introduces a potential for bias. Although the questions were reviewed by an expert panel, future studies should use a question set that has been validated by an independent committee of experts. Second, the study was conducted using a fixed set of questions, which may not fully capture the capabilities of the LLMs in a real-world setting. The performance of these models in interactive, conversational scenarios, or their ability to handle complex, case-based reasoning, remains unevaluated. Future research should explore the performance of these models in more interactive and case-based scenarios. Third, the study was conducted in May-June 2025, and the performance of the LLMs may have changed since then. Given the rapid pace of advancement in AI, the performance of the evaluated LLMs may have already changed. Acknowledging this limitation and suggesting the need for ongoing evaluation would be a valuable addition to the discussion. Finally, the figure presenting the agreement of the LLMs with expert consensus is of low resolution. A higher resolution version should be provided for publication.

It is crucial to integrate AI into surgical training courses, both to familiarize young surgeons with its capabilities and limitations, and to encourage senior surgeons to consider AI technologies, including LLMs, as accessible resources for trainees. Such integration must go hand-in-hand with robust quality control, ensuring that the knowledge gained is evidence-based and clinically sound [32]. Training programs should therefore emphasize not only what LLMs can provide but also how to critically evaluate, verify, and apply their outputs in a safe and responsible manner, with consideration of recent research findings to reinforce their applicability and limitations in clinical decision-making [31].

## Conclusion

LLMs show measurable potential as supplementary tools in bariatric surgery education, particularly for reinforcing foundational knowledge and stimulating clinical reasoning. Across 100 evidence-based questions, performance was strongest in domains anchored in well-established guidelines, such as indications, contraindications, and complication management, while accuracy dropped in areas where variability in clinical practice is greater, including preoperative preparation and postoperative care. Although newer-generation models, such as ChatGPT-4o and Gemini, demonstrated higher accuracy and moderate agreement with expert consensus, the margins of improvement over earlier models were modest, and no single model consistently outperformed across all domains. Future work should focus on refining LLM training for medical content, developing safeguards to ensure factual reliability, and exploring their role in practical, case-based, and image-driven learning contexts. Until such advances are achieved, LLMs remain promising but imperfect allies in the pursuit of scalable, evidence-based, and accessible bariatric surgery education.

## Supplementary Information

Below is the link to the electronic supplementary material.

Supplementary Material 1 Sup. 1: Full set of questions, organized into six thematic domains, used to evaluate the performance of the Large Language Models (LLMs).

## Data Availability

No datasets were generated or analysed during the current study.

## Abbreviations

AI: Artificial Intelligence
IFSO: International Federation for the Surgery of Obesity and Metabolic Disorders
κ: Kappa statistic
LLM(s): Large Language Model(s)
MCQ(s): Multiple-Choice Question(s)
MBS: Metabolic Bariatric Surgery
TAM: Technology Acceptance Model
