# Supplementary material for: Performance of Large Language Models in Metabolic Bariatric Surgery: a Comparative Study
Source: Obes Surg. 2025 Dec 11;36(2):538–45. doi: 10.1007/s11695-025-08418-y (PMC12957009; doi:10.1007/s11695-025-08418-y)
Supplement: Supplementary file 1 — Supplementary Material 1 Sup. 1: Full set of questions, organized into six thematic domains, used to evaluate the performance of the Large Language Models (LLMs). [file 11695_2025_8418_MOESM1_ESM.docx]

| Category | Q No. | Question | Options |
| --- | --- | --- | --- |
| Surgical Techniques and Procedures (n=30) | 1 | Preferred technique for bariatric surgery | Open, Laparoscopic |
|  | 2 | Drain insertion in sleeve gastrectomy is mandatory | True, False |
|  | 3 | Small mass at lesser curvature during sleeve (not in pre-op workup), best option | Complete sleeve, RYGB, OAGB, Abort surgery |
|  | 4 | 1 cm fundic lesion away from hiatus during sleeve, decision | Abort, Convert to RYGB, Convert to OAGB, Continue sleeve |
|  | 5 | Most widely used bougie size in sleeve gastrectomy | 32 fr, 36 fr, 50 fr |
|  | 6 | Most common bariatric surgery worldwide (last IFSO report) | LAGB, Sleeve, RYGB, OAGB |
|  | 7 | What is the ideal bariatric surgery | LAGB, Sleeve, OAGB, No ideal surgery |
|  | 8 | Ideal choice for GERD post sleeve | LAGB, OAGB, RYGB, Re-sleeve |
|  | 9 | Most common bariatric surgery worldwide is LAGB | True, False |
|  | 10 | Bariatric surgery under regional anesthesia | Yes, No |
|  | 11 | Gastric sleeve is reversible | True, False |
|  | 12 | Which surgery is more potent on 10 years follow-up | Balloon, LAGB, RYGB, Sleeve |
|  | 13 | Surgery to optimize effect of previous procedure named | Conversion, Revision, Redo |
|  | 14 | Length of roux limb in RYGB | 50, 100, 150, 200 cm |
|  | 15 | Length of BPL in classical RYGB | 50, 100, 150, 200 cm |
|  | 16 | RYGB is irreversible | True, False |
|  | 17 | Length of BPL in OAGB | 50, 100, 150, 250 cm |
|  | 18 | Bari-clip includes fundus resection | True, False |
|  | 19 | VBG most common in Asia | True, False |
|  | 20 | Ideal revision after VBG | Sleeve, LAGB, OAGB, RYGB |
|  | 21 | SASI approved by IFSO | True, False |
|  | 22 | There is an ideal trocar position | True, False |
|  | 23 | IFSO 2024 congress location | Germany, India, Australia, South Africa |
|  | 24 | No. of IFSO chapters | 3, 4, 5, 6 |
|  | 25 | First gastric bypass year | 1950, 1958, 1967, 1974 |
|  | 26 | % of LAGB needing revision | 20, 40, 60, 80% |
|  | 27 | Which is common as patients | Men, Women |
|  | 28 | Trade name of semaglutide | Saxenda, Mounjaro, Ozimpec |
|  | 29 | Liraglutide is GLP-1 analogue | True, False |
|  | 30 | Intragastric balloon BMI indication | 30–35, 35–40, 40–50, ≥50 |

| Category | Q No. | Question | Options |
| --- | --- | --- | --- |
| Indications & Contraindications (n=15) | 31 | Bariatric surgery not recommended in benign intracranial HTN | Yes, No |
|  | 32 | Organ transplant is contraindication | Yes, No |
|  | 33 | Optimal time post-PCI | 3m, 6m, 9m, 1y |
|  | 34 | Amputees eligible? | Yes, No |
|  | 35 | IBD patients eligible? | Yes, No |
|  | 36 | Rheumatological disease contraindication | True, False |
|  | 37 | BMI >30 → surgery not indicated (IFSO 2022) | True, False |
|  | 38 | BMI >35, no comorbidities → surgery indicated | True, False |
|  | 39 | Asians with BMI >25 can undergo surgery | Yes, No |
|  | 40 | Autoimmune disease contraindication | True, False |
|  | 41 | Bariatric surgery can trigger autoimmune | True, False |
|  | 42 | Gallstones on pre-op US → decision | Medical after, Concomitant, Delayed |
|  | 43 | Routine cholecystectomy without stones | Yes, No |
|  | 44 | Bariatric surgery decreases pregnancy incidence | True, False |
|  | 45 | Stop anti-obesity meds before surgery | 1w, 2w, 1m, 2m |

| Category | Q No. | Question | Options |
| --- | --- | --- | --- |
| Effects and Outcomes (n=15) | 46 | Can patients stop meds post-surgery | Yes, No |
|  | 47 | Bariatric surgery leads to hypertension | True, False |
|  | 48 | Cure for T2DM | Yes, No |
|  | 49 | Effect on cholesterol | ↑, ↓ |
|  | 50 | Effect on OSA | ↑, ↓ |
|  | 51 | Effect on insulin resistance | ↑, ↓ |
|  | 52 | Improve urine incontinence | Yes, No |
|  | 53 | Hair status post-surgery | Hair fall ↑, Improved, No effect |
|  | 54 | Skin changes post-surgery | Improved, Collagen ↑, More loose |
|  | 55 | New allergies post-surgery | Yes, No |
|  | 56 | Bowel movement changes | Diarrhea, No change, Constipation |
|  | 57 | Early menopause risk | Yes, No |
|  | 58 | Depression risk | ↑, ↓ |
|  | 59 | Joint pain | ↑, ↓ |
|  | 60 | PCOS after surgery | ↑, Improved |

| Category | Q No. | Question | Options |
| --- | --- | --- | --- |
| Preoperative Preparation (n=5) | 61 | Food to avoid pre-op | Carbs, Proteins, Fats, Multivitamins |
|  | 62 | Stop cannabis before surgery | 1w, 6w, 3m, 6m |
|  | 63 | IUD management before surgery | Remove, Keep, Add method |
|  | 64 | Pre-op diet mandatory | True, False |
|  | 65 | Anticoagulant prophylaxis unnecessary | True, False |

| Category | Q No. | Question | Options |
| --- | --- | --- | --- |
| Postoperative Care (n=25) | 66 | Sick leave duration | 3d, 1w, 1m, Depends |
|  | 67 | Multivitamins required? | Yes, No |
|  | 68 | Plastic surgery necessary? | Yes, No |
|  | 69 | Avoid caffeine for | 1w, 1m, 3m |
|  | 70 | Nausea duration post-sleeve | Few days, 2w, 1m |
|  | 71 | Avoid alcohol for | 1w, 3w, 3m |
|  | 72 | Hospital stay post-sleeve | 1d, 2d, 3d, Variable |
|  | 73 | Avoid side-sleeping for | 2w, 6w, 3m |
|  | 74 | Avoid driving for | 1w, 3w, 6w, 12w |
|  | 75 | Laxatives not recommended | True, False |
|  | 76 | Waist trainer recommended | Yes, No |
|  | 77 | Time to gym | 1w, 1m, 3m, 6m |
|  | 78 | Smoking allowed | Yes, No |
|  | 79 | Keto diet allowed | Yes, No |
|  | 80 | Vitamins after bypass | 6m, 1y, Life, Individual |
|  | 81 | Chewing gum allowed | Yes, No |
|  | 82 | Appetite suppressants allowed | Yes, No |
|  | 83 | Oral hypoglycemics post-op | ↑dose, Stop, Same, ↓dose |
|  | 84 | Hair fall peak | 0–3m, 3–6m, 6–9m, 9–12m |
|  | 85 | % weight loss after RYGB in 1y | 50, 60, 70, 80% |
|  | 86 | Most weight loss occurs in | 6m, 12m, 18m, 3y |
|  | 87 | Carbonated drinks allowed | 6m, Never, 1y |
|  | 88 | More lethal: Obesity vs Surgery | Obesity, Surgery |
|  | 89 | Peripheral neuropathy cause | Ca, B12, Iron, Biotin |
|  | 90 | Risk of lactose intolerance | ↑, ↓ |

| Category | Q No. | Question | Options |
| --- | --- | --- | --- |
| Complications & Management (n=10) | 91 | Cause of delayed dizziness | B12 tox, Dehydration, Ear, Not related |
|  | 92 | Hemoptysis common post-op | True, False |
|  | 93 | Pulmonary embolism common | True, False |
|  | 94 | Steatorrhea after bypass | Common, Rare, Never |
|  | 95 | Dumping more common after | Balloon, LAGB, Sleeve, RYGB |
|  | 96 | Dumping can occur after sleeve | True, False |
|  | 97 | Balloon migration with obstruction | Endoscopic, Laparoscopic, Open, Conservative |
|  | 98 | Balloon with air under diaphragm | Emergency lap, Conservative, Endoscopic, Pig tail |
|  | 99 | Gastric leak (stable patient) | Open, Stent, Conservative, Pig tail |
|  | 100 | HR 140 + Hb drop 6h postop | Conservative, Transfusion, Endoscopic, Emergency lap |
